# Incision of the pre-Descemet layer and Descemet membrane affects outflow facility: *ex-vivo* studies on a human eye perfusion model

**DOI:** 10.3389/fmed.2026.1706969

**Published:** 2026-02-06

**Authors:** Prity Sahay, Burçin Kepez Yildiz, Perla Filippini, Imran Masood, Harminder Singh Dua

**Affiliations:** 1 Academic Ophthalmology, Mental Health and Clinical Neurosciences, School of Medicine, University of Nottingham, Nottingham, United Kingdom; 2 Department of Ophthalmology, University of Health Sciences Cam and Sakura City Hospital, Istanbul, Türkiye; 3 Birmingham Midlands Eye Centre, Birmingham, United Kingdom; 4 Biomedical Sciences, University of Birmingham, Birmingham, United Kingdom

**Keywords:** Descemet membrane, Glaucoma, pre-Descemet layer or Dua layer, raised pressure, trabecular meshwork

## Abstract

**Purpose:**

The collagen and elastin fibres of the pre-Descemet layer or Dua layer (PDL) fan out to become the beams of the trabecular meshwork (TM), indicating similarities in the structural elements in the PDL and TM. This study evaluated the effects of disruption of the PDL and Descemet membrane (DM) on the facility of aqueous outflow from the anterior chamber, using human eyes and an anterior segment perfusion model (ASPM).

**Methods:**

The ASPM was established using human donor eyes. The PDL and DM in the experimental eyes were half incised circumferentially along an 8 mm diameter circle for 180°, and the other half was left non-incised.

**Results:**

The experimental eyes in the ASPM model exhibited a decrease in the outflow rate, indicating an increased outflow resistance. In the TM, the expressions of fibronectin, myocilin and alpha-smooth muscle actin were increased in incised compared to non-incised and control samples. The outflow perfusates in the experimental eyes showed a significant increase in the level of active MMP-9 on day 4 and a subsequent decrease on day 9 compared to the control eyes. Myocilin was only detected in the perfusate on day 9 in the experimental eyes.

**Conclusion:**

Our study suggests that surgical disruption of the PDL and DM results in decreased outflow, which can be due to an alteration in biomechanical properties, with consequent molecular changes in the TM, adversely affecting the resistance to outflow. This could explain why the incidence of raised pressure is greater with penetrating keratoplasty compared to deep anterior lamellar keratoplasty.

## 1 Introduction

Traditionally, penetrating keratoplasty (PKP) or full-thickness grafts have been the mainstay in corneal transplantation for more than 100 years. In the last few decades, lamellar keratoplasty, both anterior and posterior, has come to the forefront, enabling component corneal transplantation. When the stroma is diseased, it can be replaced while retaining the recipient PDL, Descemet membrane (DM) and endothelium in a procedure termed deep anterior lamellar keratoplasty (DALK). Conversely, when the endothelium is diseased, it can be replaced by selective endothelial keratoplasty wherein the diseased endothelium is stripped off and replaced by healthy donor DM and endothelium (Descemet membrane endothelial keratoplasty [DMEK]) or variations of this technique, collectively termed endothelial keratoplasty (EK) (1, 2).

Glaucoma/ocular hypertension is a known complication associated with keratoplasty (3–7), and the risk of graft failure increases if the eye pressure is high during the perioperative period (8–10). Moreover, the incidence of increased pressure is greater after PKP compared to DALK. In 2013, Dua et al. demonstrated, contrary to the prevailing belief that DALK by the big bubble technique, was not a DM baring procedure but rather a defined layer of stroma, the pre-Descemet layer (Dua layer, PDL), was retained in addition to DM in the majority of cases during DALK (11, 12). It has also been shown that the peripheral 350 microns of the PDL anterior to the DM are populated by trabecular cells that attach to the basement membrane covering the collagen beams that go on to become the core of the trabecular meshwork (TM) (13). Lewis et al. (14) and White et al. (15) demonstrated an extensive network of elastic fibres in the posterior cornea, which is maximally concentrated at the limbus as an annulus and in the PDL. They also reported that the TM extends into the posterior cornea. The periphery of the PDL therefore demonstrates homology with the TM, which has type 1 and VI collagen, elastic fibres, and a covering of the basement membrane to which trabecular cells attach.

Based on the above findings, we hypothesized that the PDL and TM act as an integrated unit and that disruption of the PDL during PKP, but not during DALK, disrupts the biomechanical equilibrium, resulting in biochemical and physiological alterations in the TM, adversely affecting its function and causing the pressure to rise.

## 2 Methods

### 2.1 Anterior segment perfusion model (ASPM)

**Figure 1:**
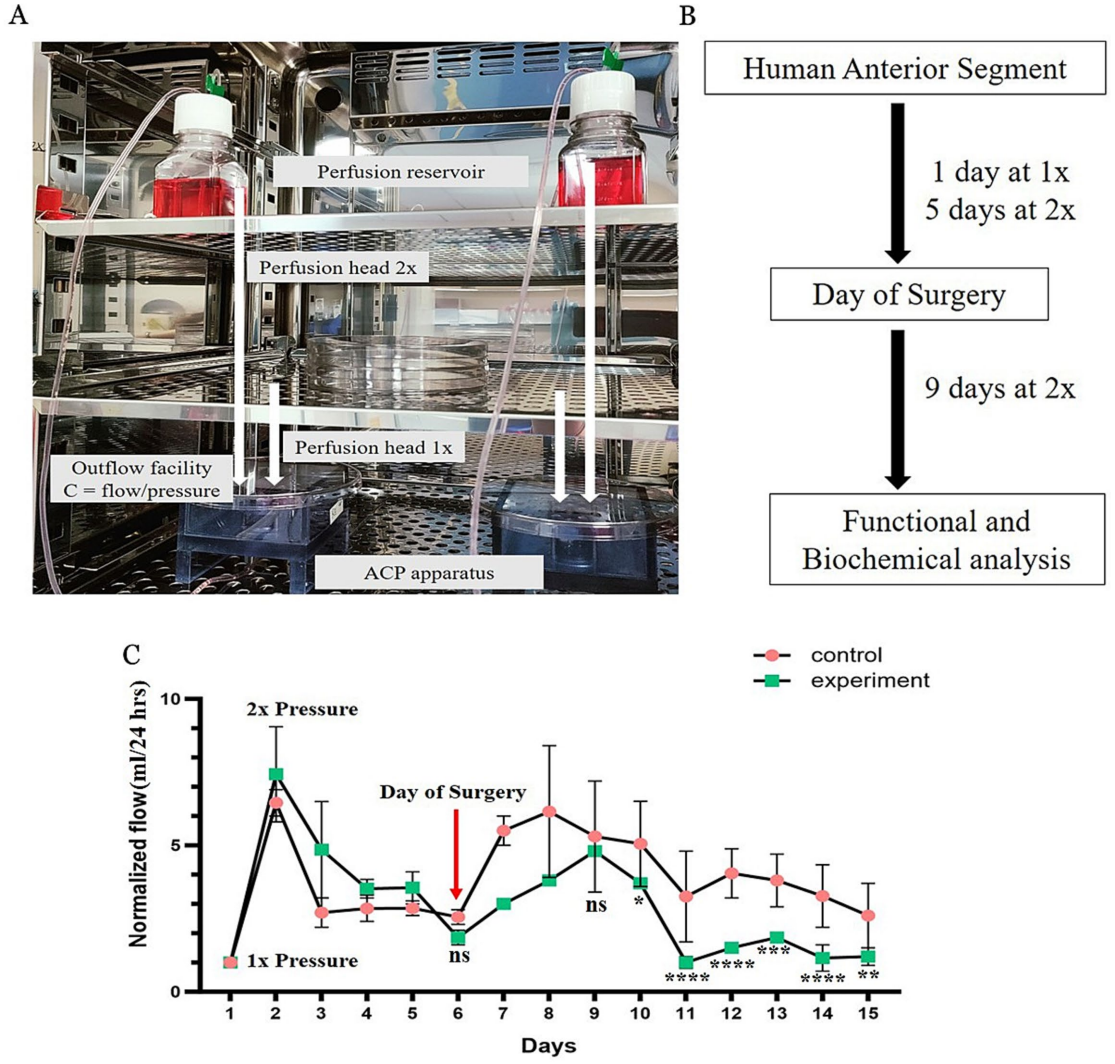
Effects of pre-Descemet layer (PDL) and Descemet membrane (DM) incisions in human donor corneas. **(A)** The anterior segment perfusion model (ASPM) used to study the outflow facility. The human anterior segment from the donor whole eye was mounted on the fluid cell of the ASPM apparatus. The short white arrow represents the distance from the ASPM apparatus to the perfusion reservoir and is termed the perfusion head 1×. A constant pressure of 8.5 mmHg was maintained for 24 h. The long white arrow represents the distance from the ACPM apparatus to the perfusion reservoir and is termed the perfusion head 2×. The perfusion head was increased to 17 mmHg for 5 days. A white tube attached to the ASPM apparatus and perfusion reservoir measures the outflow facility. **(B)** Schematic of the experimental workflow. **(C)** The experimental and control donor eyes were dismounted from the ASP, the PDL + DM of the experimental eyes were incised on day 6, and the eyes remounted on the ASP. Perfusion of all donor eyes was normalized to 2× perfusion pressure. The means ± SDs of the outflow for each day are shown. The outflow was significantly reduced in the experimental eyes compared to control for each time point after day 9. *N* = 4 for experimental eyes and *n* = 4 for control eyes from 4 different donors. One eye from the same donor served as an experimental eye, and the other eye as a control eye. ^*^p < 0.05, ^**^p < 0.01, ^***^p < 0.001, and ^****^p < 0.0001 (two-way ANOVA with Tukey’s multiple comparison test), ns, non-significant.

The ASPM was set up following previously described established protocols with some modifications (16–18). Four pairs (right and left) of human donor whole eyes (*n* = 8 eyes) were obtained from the National Health Service Blood and Transplant Service (NHSBT), Liverpool, United Kingdom. The use of whole human eyes was covered under the Human Tissue Act Licence (HTA-12265). This study was approved by the East Midlands - Nottingham 1 Research Ethics Committee (06/Q2403/46). Informed consent was obtained from all the participants. All experiments were performed in accordance with and adherence to the tenets of the Declaration of Helsinki. The demographics of the donor eyes are reported in Table 1. Human eyes were incubated in TM growth medium (low-glucose Dulbecco’s modified Eagle’s medium (DMEM) + 5% dextran + 1× antibiotic-antimycotic mixture) without serum for 5 days to facilitate recovery from postmortem storage (19), before they were mounted in the anterior segment perfusion apparatus (ASP). On the day of mounting, the posterior segment was removed from the eyes by dissecting the eyes approximately 10 mm away from the limbus in all quadrants. The anterior segment was thoroughly washed with 1× phosphate-buffered saline (PBS), and all uveal tissue remnants were removed. The anterior segment was then mounted on the ASP apparatus (custom made from Dr. Mary Kelley’s laboratory, Casey Eye Institute, Oregon, USA) and optimised by perfusing with TM culture media (low-glucose DMEM + 5% fetal bovine serum + 5% dextran + 1× antibiotic-antimycotic mixture) via a graduated reservoir (bottle) containing culture medium, placed on a sensitive weighting scale and connected to the ASP with sterile tubing. The initial bottle height was set to maintain a pressure of 8.5 mmHg (termed 1× pressure) for 24 h and followed by perfusion at 17 mmHg (2× pressure) by raising the perfusion reservoir to the appropriate height, for additional 5 days as previously published (16). An illustration of ASPM is shown in Figure 1A.

**Table 1:** Demographics of the human donors’ eyes.

Donor identifier code	Age	Sex	Post-mortem times
G170023306708G OD	70	M	11 h 58 min
G170023306708G OS	70	M	11 h 58 min
G1700233068512 OD	91	F	23 h 8 min
G1700233068512 OS	91	F	23 h 8 min
G170023307726X OD	90	F	11 h 25 min
G170023307726X OS	90	F	11 h 25 min
G1700233082912 OD	78	M	19 h 10 min
G1700233082912 OS	78	M	19 h 10 min

One eye of each pair was used as the control.

In the experimental eyes, one eye from each donor pair (*n* = 4 donor human eyes), the PDL and DM were incised from the endothelial surface, 35–40 microns deep and 2–2.5 mm inside the internal limbus, corresponding to an 8 mm graft, along a 180° semicircle with a calibrated angled adjustable diamond blade (Katena, Corza medical, Parsippany, New Jersey, United States) (termed the incised half), leaving the other 180° intact (termed the non-incised half). The other eyes of each pair (*n* = 4 donor human eyes) served as controls. The control eyes were demounted and remounted as the experimental eyes at the time of incision, without any incision. The surgically incised experimental and control eyes were again placed on the ASP with the TM culture medium and perfused at the homeostatic pressure of 17 mmHg (2×). An experimental schematic of the workflow is given in Figure 1B. Flow rates were measured by weighing the reservoir after 24 h and normalizing it to the initial baseline weight by topping up the fluid of the perfusion reservoir.

### 2.2 Sample collection after perfusion

Each day, the perfusate was collected from the perfusion reservoir in the ASP apparatus and stored at −80 °C until future biochemical analysis. After the perfusion experiment, tissue samples of the TM and peripheral cornea, from the experimental (including samples from incised and non-incised halves) and control groups were obtained. The samples were fixed with 4% paraformaldehyde (PFA) at 4 °C for 20–30 min at room temperature (RT). The samples were subsequently incubated overnight in 30% sucrose at 4 °C, embedded in an optimal cutting temperature compound (OCT) cryoembedding matrix, and stored at −80 °C before cryosectioning. Tissue sections from each sample were stained with hematoxylin and eosin (H&E) to verify a break in the PDL/DM where the incisions were made and the corresponding intact PDL/DM where incisions were not made, and in the controls (Supplementary Figure 1).

### 2.3 Immunohistochemistry

The OCT cryo-embedded matrix was cut into 10 μM sections, rehydrated in 1× PBS at RT for 5 min, and permeabilized with 0.1% Triton X-100 in PBS for 15 min. The sections were then blocked with 10% normal goat serum for 1 h at RT and sequentially incubated overnight with specific primary antibodies against fibronectin (FN) at 1:100 (ab2413, Abcam), alpha smooth muscle actin (*α*-SMA) at 1:50 (ab7817, Abcam), collagen-IV at 1:50 (ab86042, Abcam), myocilin at 1:50 (ab41552, Abcam), and vimentin at 1:200 (ab92547, Abcam) in 5% normal goat serum at 4 °C. Each section was washed with PBS three times and then incubated with the corresponding fluorescent dye-conjugated secondary antibodies, including anti-rabbit Alexa Fluor 488 at 1:1,000 (ab150081, Abcam) and anti-mouse Alexa Fluor 594 at 1:1,000 (ab150120, Abcam), in 5% normal goat serum at RT for 1 h in the dark. After three washes with 1× PBS for 5 min each, the sections were mounted with mounting medium (ProLong Diamond Antifade Mountant, Invitrogen, USA) and a coverslip. The sections were imaged using a fluorescence microscope (Leica Microsystem CMS GmbH; Model DMIL LED Fluo; Wetzlar, Germany). The images were analysed in Leica Application Suite X (3.0.0.15697), and the composites were prepared using Adobe Photoshop CS5 (Adobe Systems, San Jose, CA). ImageJ software (http://imagej.nih.gov/ij/; provided in the public domain by the National Institutes of Health [NIH], Bethesda, MD, USA) was used to obtain the mean fluorescence intensity of the proteins studied, which was expressed in arbitrary units (AUs). The laser settings and acquisition parameters; exposure times (500 ms), and gain (1), were maintained the same for all immunofluorescence images. As the background intensities showed some variation (possibly related to autofluorescence) the corrected total fluorescence intensity was calculated by normalizing the selected area of fluorescence intensity with the background reading. Control immunohistology of all samples, without application of primary antibody, was carried out where appropriate.

### 2.4 Gelatin zymography

The enzymatic activity of MMP-9 and MMP-2 was examined by substrate gelatin zymography as previously described (20). A bicinchoninic acid (BCA) protein assay kit was used to quantify the total protein in the perfusate samples (Merck Millipore, United Kingdom). Equal amounts of protein (80 μg) in 4× lithium dodecyl sulphate (LDS) sample buffer (Merck Millipore, United Kingdom) were separated on 10% sodium dodecyl sulphate–polyacrylamide gel electrophoresis (SDS–PAGE) gels containing 0.1% gelatin. The gels were washed in 2.5% Triton X-100 washing buffer and then incubated in incubation buffer containing 50 mM Tris–HCl, 10 mM CaCl_2_, l M ZnCl_2,_ and 200 mM NaCl, pH 7.5, at 37 °C for 16 h. The gels were stained with Coomassie blue solution (0.05% Coomassie brilliant blue R-250 in 40% methanol and 10% acetic acid) and partially destained with destaining solution (20% methanol and 10% acetic acid) to visualize the clear zone of gelatin lysis against the blue background stain, indicating the presence of MMPs. The zymographic gels were imaged in an Odyssey LI-COR System (OFC- 0343), and lysis zones in every lane were analysed by Image Studio acquisition software in the LI-COR instrument (model number 1.0.37) to obtain band intensities with metalloproteinase 2 and 9 activities. ImageJ software was used to obtain the enzymatic activities of total and active MMP-9 and MMP-2 expressed in arbitrary units (AUs).

### 2.5 Western immunoblotting

An equal amount of protein (80 μg) was loaded per lane in 10% SDS–PAGE gels. The gels were run at 100 V for 60 min and wet transferred at 4 °C to polyvinylidene fluoride membranes. Bovine serum albumin (5%) was used as a blocking buffer. The blots were incubated with primary antibody against myocilin at 1:1,000 at 4 °C overnight. The membranes were then incubated for 1 h with IRDye-labelled (800CW) secondary antibody at 1:10,000 at room temperature. The protein bands were detected using the Odyssey Fc System before their intensities were analysed via Image Studio software (5.0, LI-COR Biotechnology, Lincoln, NE, USA).

### 2.6 Enzyme-linked immunosorbent assay (ELISA)

The concentration of tissue inhibitor of metalloproteases (TIMP-1) in the perfusate from the perfusion reservoir was analysed by colorimetric immunoassays according to the manufacturer’s instructions (ab187394, Abcam). The absorbance was measured on an ELISA microplate spectrophotometer (BMG Labtech, Ortenberg, Germany) at 450 nm.

### 2.7 Statistical analysis

GraphPad Prism 10.0.2 was used to create a grouped comparison graph. Each experimental and control eye from 4 different donors was compared for the statistical analysis and the results were presented as the means ± standard deviations (SDs). Two-way ANOVA with Tukey’s multiple comparisons test was used to calculate significance, with a *p* value of < 0.05.

## 3 Results

### 3.1 Surgical disruption impairs the flow rate in the experimental eyes

The anterior segments with incised DM and the PDL in the experimental eyes presented a gradual decrease in the outflow rate compared to control eyes when subjected to 2× pressure. There was no difference in the outflow rate in either sample on day 6, the day of incision. Thereafter, the system stabilized over days 6–9, beyond which the experimental eyes presented a significant decline in the outflow rate compared with that of the controls for every 24 h time point from day 10 to day 15, the endpoint of the experiment (Figure 1C). A table highlighting the values has been provided (Supplementary Table 1). The IOP with an iCare® tonometer ic100 (Finland Oy, Vantaa, Finland) and corneal thickness with a pachymeter, Pachmate 2 (DGH Technology, Inc., Exton, PA, USA), were also measured before and after the experiment. Before the experiment, the IOP for the control eyes was 23.6 ± 2.6 mmHg, and after the experiment, it was 20.2 ± 2.3 mmHg. Before the experiment, the IOP for the experimental eyes was 22.6 ± 0.7 mmHg, and after the experiment, it was 21.9 ± 2.7 mmHg. Similarly, before the experiment, the corneal thickness for the control eyes was 985 ± 9.1 μm, and after the experiment, it was 904 ± 1.47 μm, and before the experiment, the corneal thickness of the experimental eyes was 896.6 ± 13.4 μm, and after the experiment, it was 758 ± 27 μm. None of these differences were statistically significant.

### 3.2 Surgical disruption leads to increased levels of fibrotic proteins in the TM

**Figure 2:**
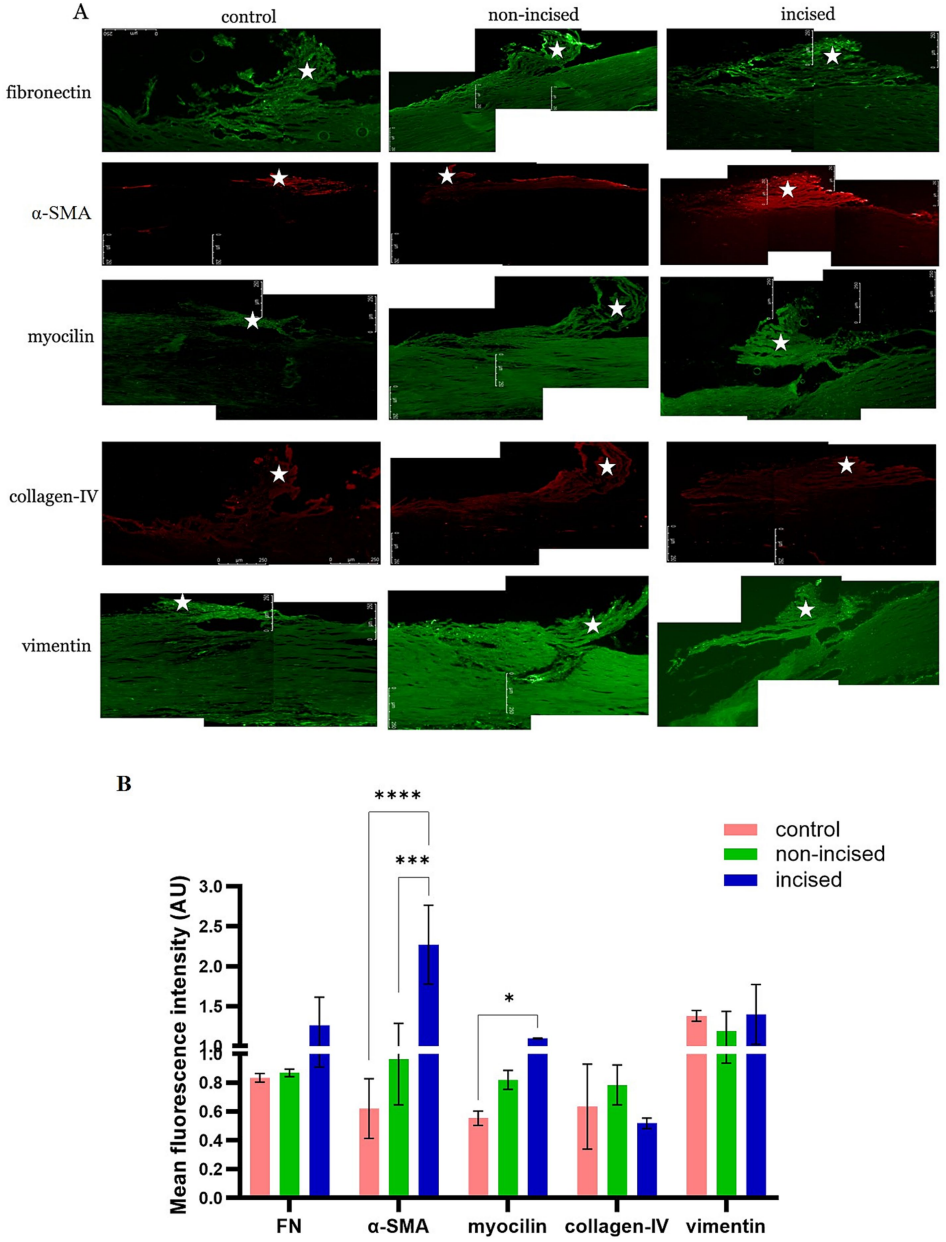
Fibrotic protein expression between incised and non-incised halves of the trabecular meshwork (TM) and controls. Immunofluorescence staining in TM (represented by *) sections from non-incised, incised, and control samples showing **(A)** comparison of the following fibrotic protein markers: fibronectin (FN), *α*-smooth muscle actin (α-SMA), myocilin, collagen-IV, and vimentin. The levels of the FN, α-SMA, and myocilin proteins were increased in incised compared to non-incised and control samples. The scale bar of all images represents 100 μm. **(B)** Densitometry analysis of the intensity of fluorescence expressed by different proteins in the 5 different locations of the TM via ImageJ software. The values are expressed as the mean fluorescence intensity ± SD. ^*^p < 0.05, ^***^p < 0.001, and ^****^p < 0.0001 (two-way ANOVA with Tukey’s multiple comparison test).

In the TM, we observed that the FN was increased in incised compared to non-incised samples and controls. Myocilin and *α*-SMA expressions were significantly increased in the incised samples than in non-incised samples compared to controls. Collagen-IV and vimentin did not show a statistically significant change in incised and non-incised samples compared to controls (Figures 2A,B). A representative image of DAPI staining with a no primary antibody staining is shown (Supplementary Figure 2).

### 3.3 Surgical disruption altered the biochemical turnover of the extracellular matrix

**Figure 3:**
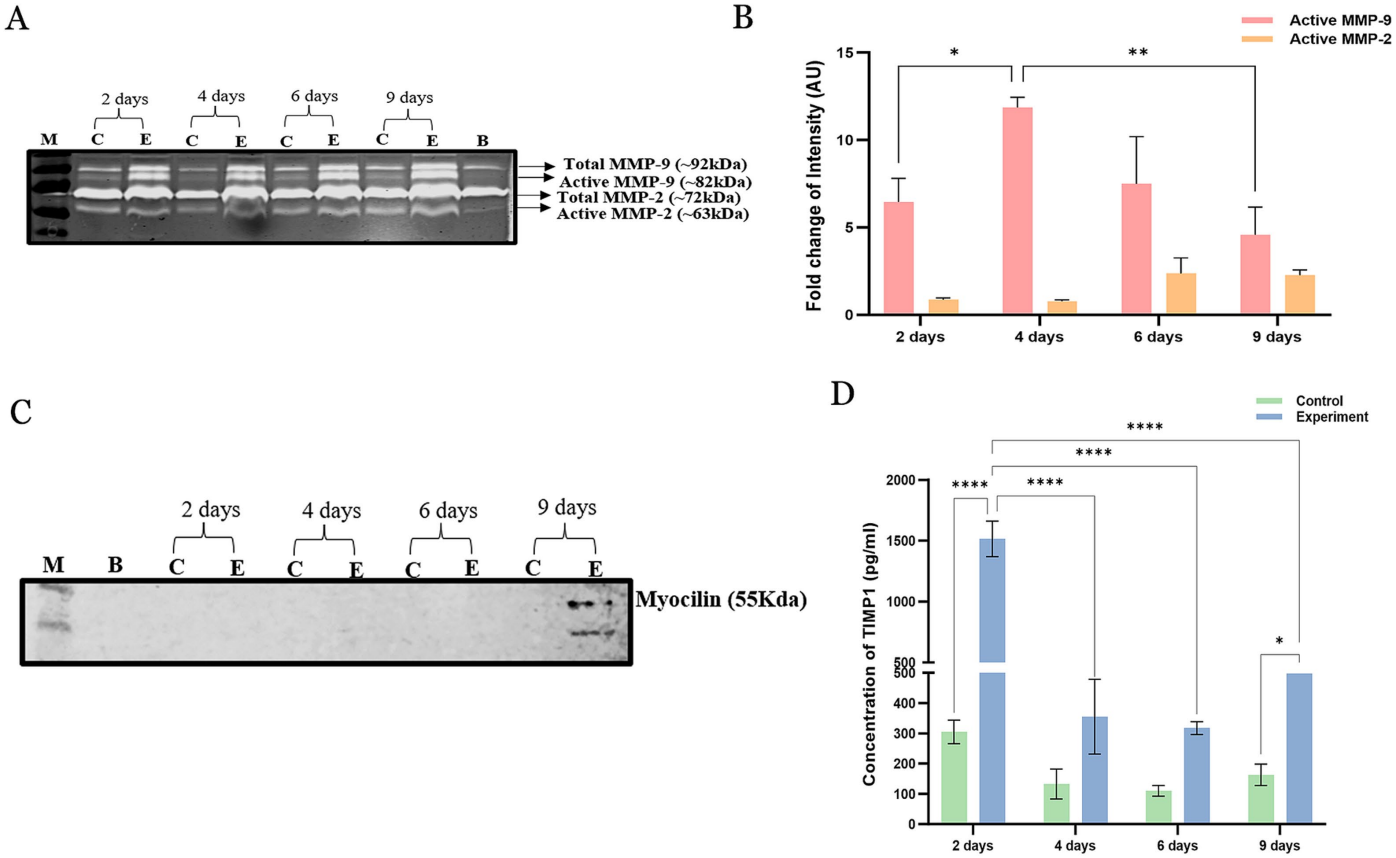
Biochemical analysis of the perfusate collected from incised and non-incised samples. **(A)** Estimation of matrix metalloproteases (MMPs)-2 and −9 by gelatin zymography. **(B)** Densitometry analysis to compare the fold changes in MMP-9 and MMP-2 expression between experimental and control perfusate samples via ImageJ software. The fold change in the levels of active MMP-9 significantly increased on day 4, then gradually decreased on day 6, and then significantly decreased on day 9 compared with that of the controls. **(C)** The immunoblot assay revealed a significant presence of the myocilin protein on day 9 of the experiment compared with the other time points and controls. **(D)** Estimation of the TIMP-1 protein concentration by ELISA. The mean TIMP-1 levels were significantly increased on day 2 post-incision than on the other days and in the control groups. The data are shown as the mean ± SD. C, control; E, experiment; M, marker; B, blank (only media); ^*^p < 0.05, ^***^p < 0.001, and ^****^p < 0.0001 (two-way ANOVA with Tukey’s multiple comparison test).

We observed that the fold change of active MMP-9 is significantly increased on day 4 post-incision, then gradually decreased on day 6, and then significantly decreased on day 9 compared to controls. In the lane 10, only culture medium was added to normalize MMPs activities in the serum and medium alone. The fold change in MMP-2 did not significantly differ between the incised samples and the control samples (Figures 3A,B). The immunoblot assay revealed a significant presence of the myocilin protein on day 9 but not before, compared with the other time points and controls (Figure 3C). Similarly, this corresponded with the increased expression of myocilin in tissue sections of the TM after the completion of the experiment. The TIMP-1 protein concentration was significantly increased on day 2 post-incision, whereas on days 4, 6, and 9, the TIMP-1 protein level was significantly lower than that on day 2 of the experiment compared with that of the controls (Figure 3D).

**Figure 4:**
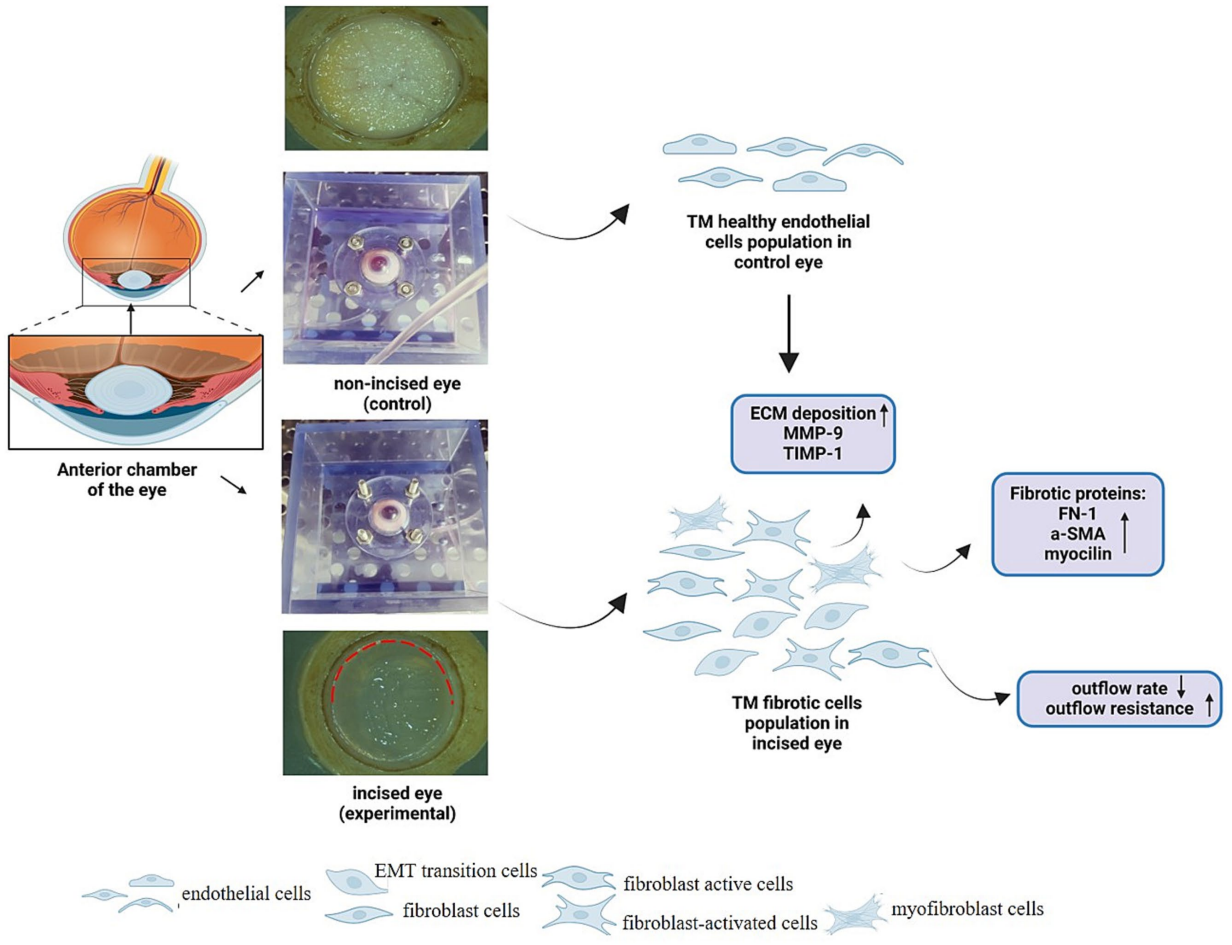
Summary and key findings. The anterior chamber of the whole human eye was used to set up the experiment in the anterior segment perfusion model. The illustration of the excised Descemet layer (PDL) and Descemet membrane (DM) (circumferentially along an 8 mm diameter circle for 180°) highlighted with red broken lines and non-excised eye in the experimental model (lower panel) and control eye (upper panel) in the anterior segment perfusion model were shown. The incision of PDL and DM in the peripheral cornea resulted in the population of myofibrotic and fibrotic endothelial trabecular meshwork (TM) cells which eventually leads to imbalance in TIMP-1/MMP-9 ratio, fibrotic proteins deposition, and compromise aqueous humor outflow. The other eye is a control eye that have a population of healthy endothelial TM cells. TIMP-1, tissue inhibitors of metalloproteinases-1; MMP-9, matrix metalloproteinases; ECM, extracellular matrix; TM, trabecular meshwork; FN, fibronectin; α-SMA, alpha-smooth muscle actin.

A summary of the key findings of the study is summarized schematically in Figure 4.

## 4 Discussion

PKP is associated with raised eye pressure/glaucoma in the early and late postoperative periods. The reported incidence ranges from 9 to 50%, ranging from 10 to 31% in the early postoperative period and from 18 to 35% in the late postoperative period (3–5). Preoperative and postoperative glaucoma after keratoplasty are important, as they are associated with an increased risk of rejection and non-rejection graft failure and optic nerve damage (8–10). The increased incidence of glaucoma after PKP is attributed to a number of factors (3–5, 8, 9) such as pre-existing glaucoma, the use of steroids, especially if the patient is a steroid responder, postoperative inflammation, the size of the graft (21), and suture-related distortion of the anterior chamber angle. A difference in IOP has been reported between PKP and DALK, which is significantly greater with PKP than with DALK (and other endothelial keratoplasty procedures) (10, 22). However, the occurrence of glaucoma after PKP is greater than what can be explained by the use of steroids alone, as the same dose of steroids at the same frequency is used in patients undergoing DALK in the first 12 weeks, although the total duration of steroid use is shorter after DALK than after PKP. Suture-related distortion of the angle is a major cause of PKP. It is suggested that sutures placed in PKP can produce a posterior wound gape, which can cause the incised DM to retract posteriorly towards the angle of the anterior chamber, undermining the anterior support that can lead to collapse of the TM and impede aqueous outflow (23).

However, the suturing technique and number of sutures used are similar for the DALK and PKP procedures, but the incidence of increased IOP is different; hence, this difference cannot be attributed to the use of sutures alone. Other variables, such as size and stroma-related indications, are also similar. In PKP, the PDL and DM are incised, unlike in DALK, where these tissues are spared. We explored this aspect by incising the DM and PDL 180° along the circle where an 8 mm PKP graft would incise them and showed that the flow rate was impaired despite the lack of sutures. This can be attributed to altered biomechanics of the TM following transection of the DM and PDL, as both structures, especially the latter, are contiguous with the TM (13, 24). The corneal thickness was spuriously increased in our donor eyes, as would be expected to occur postmortem. The increase was not different across the control and experimental eyes; however, this could have impacted the accurate measurement of the IOP, which was not found to be elevated corresponding to the reduced outflow. It is well known that inherently thick corneas give spuriously higher pressure readings related to the increased collagen lamellae and altered biomechanical properties (25, 26). Corneas that are thicker due to accumulation of fluid (edema) behave differently and confound pressure measurements. This is the most likely explanation for the higher than 2× pressure measurements obtained in our experiments, both in the postmortem study and the control eyes, where the thickness too was greater by 300–400 microns compared to normal in-vivo thickness. The inaccuracy in IOP measurement is a limitation and could be eliminated with the use of an intraocular transducer to measure IOP in future studies.

Biomechanical factors influence cell signalling, protein and gene expression in the TM (27, 28). Among the proteins involved in the maintenance of extracellular matrix homeostasis in the TM, FN was increased in the incised half of the sample compared with the non-incised halves and the controls; *α*-SMA and myocilin were significantly increased in the incised compared to the non-incised halves and controls. These findings concur with the study of Yang YF et al. who recently reported that increased expression of classic endothelial-to-mesenchymal transition biomarkers like α-SMA, FN with other biomarkers N-cadherin, Snail Family Transcriptional Repressor 2 (*SNAI2*), *vimentin*, Transforming Growth Factor-*β* (TGFβ) in glaucomatous TM cells, negatively impacts aqueous humor outflow (29). Myocilin is a secreted protein found in many normal tissues and organs, but diseases related to myocilin are believed to occur only in the eye (30). Myocilin is secreted in the TM, is present in aqueous humor (31), and importantly, has a significant role in the regulation of intraocular pressure (32). Accumulating evidence strongly supports the notion that myocilin, the TM, IOP and glaucoma are closely associated (33). The presence of myocilin in the perfusate only on the day 9 of the post-incision of DM and PDL and increased expression in tissue sections of the TM suggest that these two layers contribute to TM homeostasis, most likely through their effects on TM biomechanics. This does not seem to be a nonspecific response to the incision of the proteins studied; some were variably affected, whereas others (collagen VI and vimentin) were not. The timeline related to the increase in myocilin secretion needs to be further studied, as we only observed it on day nine post-incision, which corresponded to the end point of the experiment (day 15). The model has been validated for a total of 2 weeks from set-up (16); hence, information beyond that point could not be obtained but would be useful.

The correlation between the decreased outflow following incision strongly indicates that the disruption of the DM and PDL induced a change in outflow resistance, reducing outflow. This could therefore be an important mechanism by which increased IOP/glaucoma occurs in PKP compared with DALK, where these layers are preserved. In this study, it was not possible to determine the contribution of each layer to the reduced outflow. Many of the studies comparing DALK with PKP did not differentiate between DALK with a type-1 big bubble, where both the PDL and DM are preserved, and DALK with a type-2 big bubble (or mixed big bubble), where only the DM is preserved. Given that the different types of big-bubbles are clearly understood and can be identified intraoperatively, future studies addressing this aspect in patients undergoing DALK should be conducted. However, it has been reported that a type 1 big bubble is attained in 85% of patients undergoing DALK (34–36); Hence, it would require a multi-centre study to attain a reasonable number of cases with at type-2 big bubble to make such a comparison. Until then, the comparison between DALK and PKP would largely apply to type-1 big bubbles, where both the DM and PDL are preserved. Similarly, further studies involving a precise incision, perhaps with a femtosecond laser, cutting the DM alone, the PDL alone or both together, which should be possible at least in theory, could help tease out the impact of the individual layers.

The difference in the presence of TIMP-1, MMP-9 and MMP-2 in the perfusate of the experimental eyes versus the control eyes is also interesting. TIMP-1 showed a significant early increase (day 2) and then a decline but remained higher than that of the controls, whereas the proinflammatory active protein MMP-9 was significantly increased on day 4 and gradually decreased thereafter, although it remained above the levels detected in the control samples. This appears to be a specific response, as the expression of MMP-2 did not significantly change. The increased expression of MMP-9 could be related to the surgically induced inflammatory response, but generally suggests that metalloproteases and their inhibitors could play a role in reduced outflow following corneal surgical intervention. The initial spike in the expression of TIMP-1 in the control and the experimental eyes on day 2 post incision could be due to the effect of handling, and crushing effect of dismounting and remounting of the samples, with the addition of the incision made in the experimental eyes, where the spike was higher.

This study establishes the relationship between the posterior cornea layers and the TM in the context of the outflow facility of the TM. This is clinically relevant as it establishes a functional relationship between the PDL/DM and the TM to complement the anatomical relationship previously reported. It emphasizes the fact that during anterior lamellar keratoplasty, all attempts should be made to preserve the PDL to reduce risk of raised IOP postoperatively and improve outcome of surgery. This study lays the foundation for studies to explore the role of the PDL in the context of IOP maintenance and glaucoma. It is well known that corneal thickness and biomechanics have a significant impact on IOP, glaucoma and glaucoma related damage. Study of the precise biomechanics of the PDL is likely to provide further insights on pathophysiology of glaucoma. This study also establishes the ASPM as a useful tool for studying *ex-vivo* aspects related to intraocular pressure maintenance in the human eye.

Overall, this study shows that disruption of the PDL and DM at the corneal periphery compromises aqueous outflow, which is associated with increased deposition of fibrotic proteins and an imbalance of MMP-9/TIMP-1 ratio in the ECM of TM. This coupled with the reported anatomical demonstration that the TM is a continuation of the peripheral PDL, can explain the increased incidence of raised eye pressure in PKP compared to deep anterior lamellar keratoplasty where the PDL and DM are not incised.

## Data Availability

The raw data supporting the conclusions of this article will be made available by the authors, without undue reservation.
