# Supplementary material for: Incision of the pre-Descemet layer and Descemet membrane affects outflow facility: ex-vivo studies on a human eye perfusion model
Source: Front Med (Lausanne). 2026 Feb 6;13:1706969. doi: 10.3389/fmed.2026.1706969 (PMC12920517; doi:10.3389/fmed.2026.1706969)
Supplement: Supplementary file 1 [file Data_Sheet_1.docx]

Supplemental Figure 1. A representative image to show the control non-incised, and incised PDL+DM.


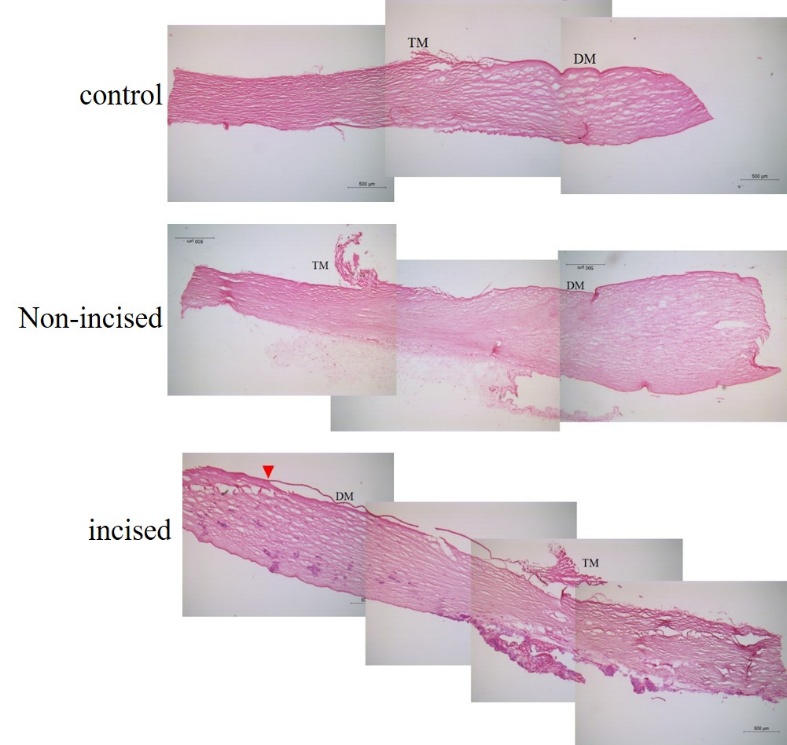


Supplemental Figure 2: A representative image of DAPI staining without the use of the primary antibody in the immunostaining procedure.


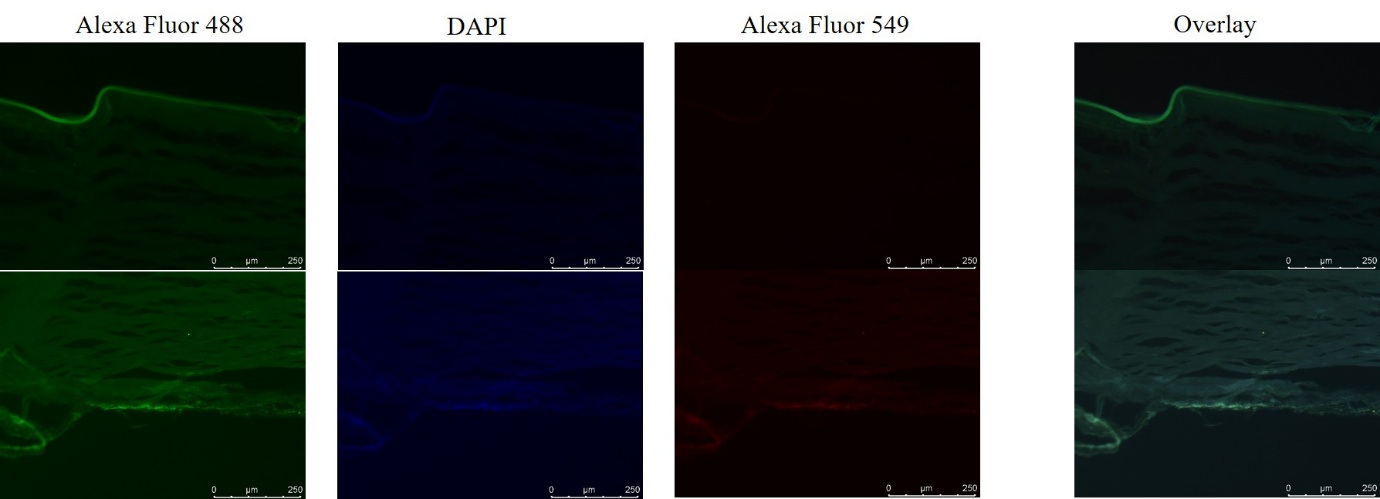


Supplemental Figure 3: Large individual images the “non-incised” eyes to illustrate the trabecular meshwork area that was analysed.

Non-incised a-SMA (R):


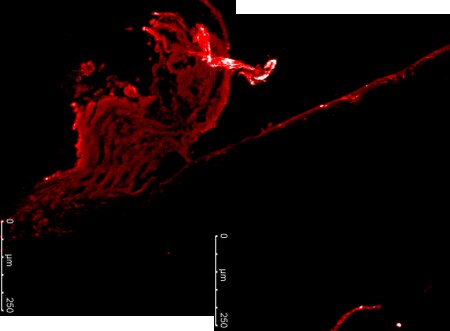


Non-incised Col-IV (R):


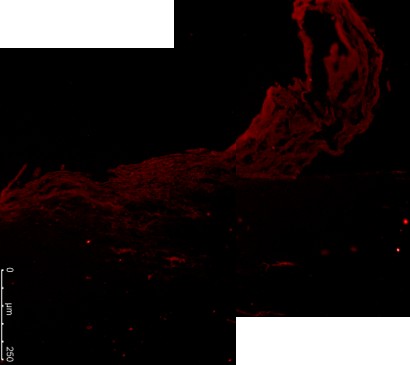


Non-incised FN (G):


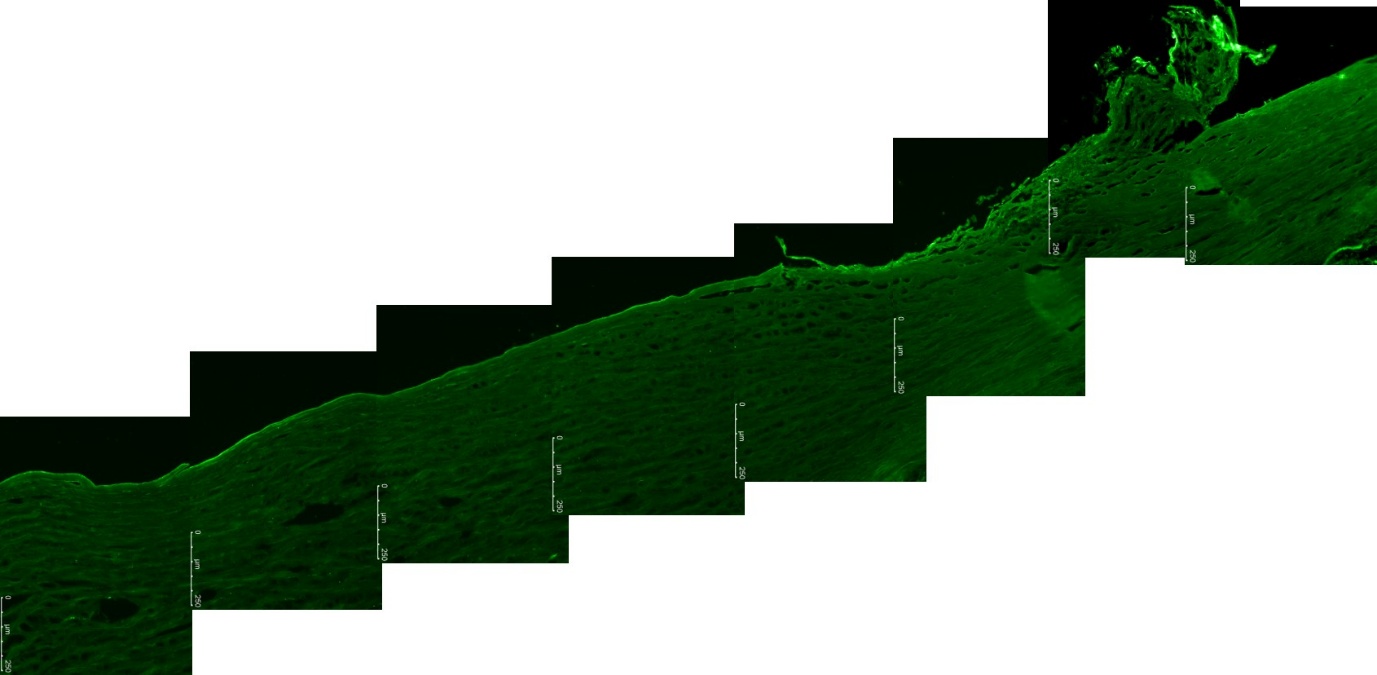


Non-incised Myocilin (G):


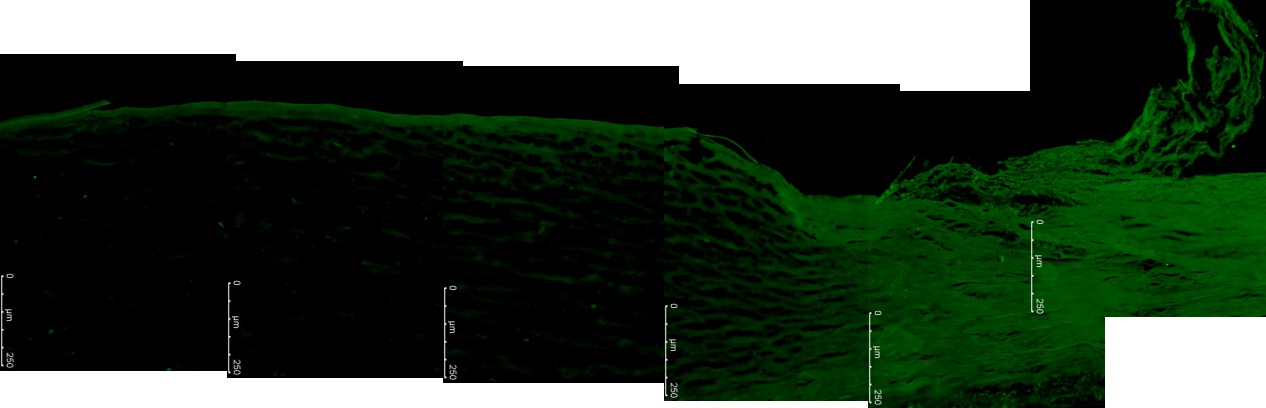


Non-incised vimentin (G):


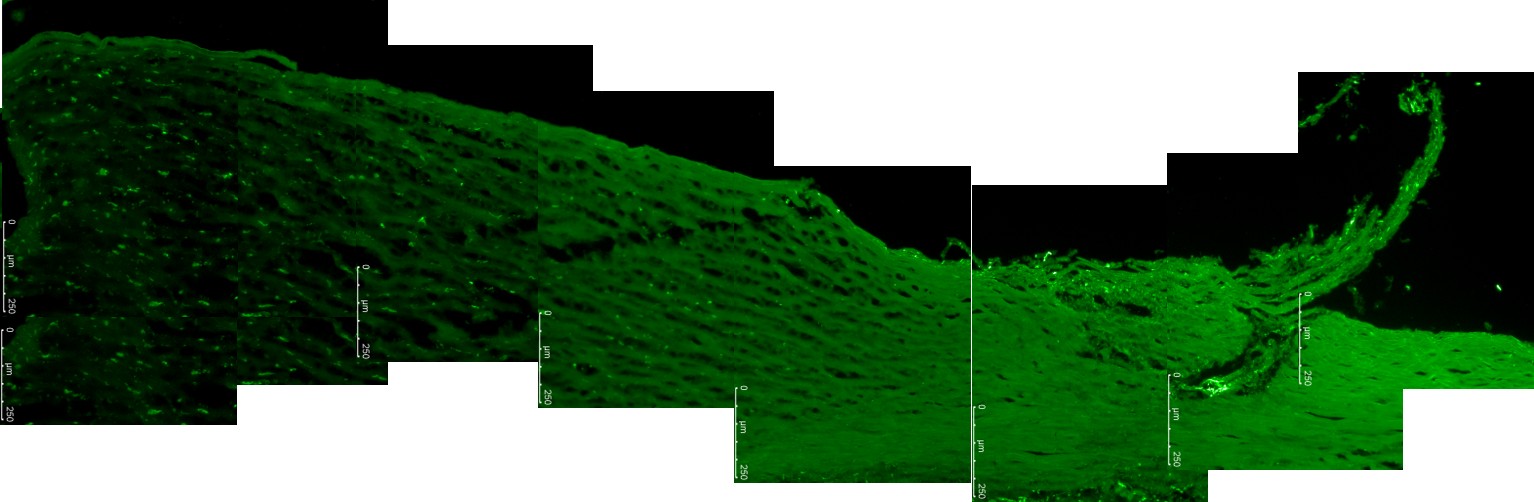


Supplemental Table 1. A table showing values of outflow in the control and experimental eyes.

| Days | Control (ml) | Experiment (ml) |
| --- | --- | --- |
| 1 | 1.0 | 1.0 |
| 2 | 6.5 | 7.5 |
| 3 | 2.75 | 4.75 |
| 4 | 3.0 | 3.75 |
| 5 | 3.25 | 3.75 |
| 6 | 2.5 | 2.0 |
| 7 | 5.5 | 3.0 |
| 8 | 6.0 | 4.0 |
| 9 | 5.5 | 5.0 |
| 10 | 5.25 | 4.0 |
| 11 | 3.5 | 1.0 |
| 12 | 4.5 | 2.0 |
| 13 | 4.25 | 2.5 |
| 14 | 4.0 | 1.5 |
| 15 | 3.25 | 1.75 |
